# Anti-atherogenic and cardio-protective properties of sweet melon (*Cucumis melo*. L. Inodorus) seed extract on high fat diet induced obesity in male wistar rats

**DOI:** 10.1186/s12906-022-03793-w

**Published:** 2022-12-20

**Authors:** G. Adebayo-Gege, V. Alicha, T. O. Omayone, S. C. Nzekwe, C. A. Irozuoke, O. A. Ojo, A. F. Ajayi

**Affiliations:** 1 Department of Human Physiology, Faculty of Basic Medical Sciences, Baze University, Jabi, Nigeria; 2 Department of Human Physiology, Faculty of Basic Medical Sciences, College of Medicine Bingham University, Jos, Nigeria; 3 Department of Human Physiology, Faculty of Basic Medical Sciences, College of Medicine, FUTA, Akure, Nigeria; 4 Department of Biochemistry, Faculty of Science, Adeleke University, Ede, Osun State Nigeria; 5 Department of Anatomy, Faculty of Basic Medical Sciences, Baze University, Jabi, Nigeria; 6 Phytomedicine, Molecular Toxicology, and Computational Biochemistry Research Laboratory (PMTCB-RL), Department of Biochemistry, Bowen University, Iwo, 232101 Nigeria; 7 Department of Physiology, Faculty of Basic Medical Sciences, College of Health Sciences, Ladoke Akintola University of Technology, Ogbomosho, Oyo State, Nigeria; 8 Anchor BioMed Researh Institute, Ogbomoso, Oyo State Nigeria

**Keywords:** Sweet melon seeds, Atherogenic index, Obesity, Nitric oxide, Oxidative stress

## Abstract

**Background:**

Cucumis melon is a medicinal plant with multiple pharmacological properties such as anti-inflammatory, antioxidant, and diuretic effects. An increasing body of scientific evidence established the anti-diabetic/anti-obesity effects of *Cucumis melo* in humans, mice, and hamster models. However, there are no tangible reports on its ability to prevent cardiovascular complications following diet-induced obesity. The anti-atherogenic and cardioprotective effects of the Methanolic extract of *Cucumis melo*. L. Inodorus seeds on a high-fat diet (HFD)-induced obese rats was assessed in this study.

**Methods:**

Forty male Wistar rats were randomly divided into five groups, (*n* = 8/group); i.e., Normal (N), HFD, HFD + 50 mg/kg b.w. of MCMs (Methanolic extract of *Cucumis melon* seeds), HFD + 100 mg/Kg b.w. of MCMs and HFD + 200 mg/kg b.w. of MCMs. The experimental animals were anaesthetized and sacrificed after 10 weeks, and blood samples and heart tissue were collected for further analysis. Using the Graph Pad Prism version 5.0, the results expressed as Mean ± SD was tested using the one-way ANOVA to show intergroup differences, followed by Bonferonni ‘s post hoc test. The level of significance was determined at *P* ≤ 0.05.

**Results:**

MCMs significantly (*P* < 0.05) reduced body weight, adiposity index, total fat mass, low-density lipoprotein cholesterol (LDL-c), and total cholesterol (TC) compared with the HFD obese groups MCMs caused a significant reduction in the body weight, total fat mass, adiposity index, low-density lipoprotein cholesterol (LDL-c), and total cholesterol (TC) when compared to the animals in HFD obese groups. Also, the Atherogenic index of plasma (AIP), Castelli index and, malondialdehyde (MDA) significantly (*P* < 0.05) decreased in MCMs treated groups compared to the HFD obese group. The catalase, protein, and HDL levels were significantly increased in MCMs treated groups compared to HFD-obese animals. Expression of nitric oxide in the form of nitrite in the heart tissue significantly increased in the MCMs treated compared to the HFD-obese rats, with the majority of the positive results recorded at 100 mg/Kg b.w. of MCMs.

**Conclusions:**

MCMs have anti-atherogenic and Cardio-protective properties on High Fat Diet-Induced Obesity in Male rats via an antioxidant and nitric oxide-dependent mechanism. Further study is recommended to evaluate the molecular mechanisms to which these anti-atherogenic and cardio-protective actions can be attributed and exploit the GCMS result in the development of drug candidates.

## Background

Obesity is a medical condition characterized by excess accumulation of fat in different parts of the body or its organs which results in adverse effects on the health of an individual [1]. Obesity is said to have occurred when the body mass index (BMI), which is the ratio of a person’s weight to the person’s height squared is greater than 30 kg/m2 [1]. Combined factors like sedentary lifestyle, excessive food intake, and genetic susceptibility frequently cause obesity [2]. Endocrine disruptions, side effects of medications, or mental disorders have been reported as primary causes of some cases of Obesity [3].

There have been a significant increase in the instances of obesity and associated disorders in the last decades, and this poses a considerable risk to the human health [4]. About 36% of the human population is obese, and if not well addressed, approximately 1 billion of the adult population may become obese by 2030. All the factors predisposing people to obesity also lead to an increase in the deposition of adipose tissues in the different organs of the body, the adipose tissue composed mostly of adipocytes [5]. Due to a difference in the sex hormones produced by males and females, the sites of fat storage in both males and females are different. There is a higher tendency of fat deposition and storage around visceral organs in men, and this leads to obesity around the middle part of their abdomen. Conversely, women tend to store more fat subcutaneously within the gluteal and thigh region [5]. Most times the risk of cardiovascular complications, which include the development of hypertension, coronary artery disease, and metabolic abnormalities, is increased by the presence of excess adipose tissue, mainly in the abdomen [6].

The main treatments for obesity are changes to diet and exercise [7]. Reduction of energy-dense food, including foods with high sugar and fat and an increase in the intake of dietary fibre, is an important dietary recommendation that can help with the management of obesity [1]. Appetite and fat absorption can also be reduced by the use of medications too [8]. Gastric balloon or Surgery (to reduce stomach volume or intestine length) are usually considered in cases where exercise, diet, and medication have proven to be ineffective [9, 10]

Lipid-rich diet has been reported to possess the ability to generate ROS by altering oxygen metabolism; also, an increase in deposition of adipose tissue can result in a decrease in the activity of antioxidant enzymes like glutathione peroxidase (GPx), catalase (CAT), superoxide dismutase (SOD). Various abnormalities can result from the increase in ROS production and the decrease in antioxidant activities. These abnormalities include endothelial dysfunction, where the bioavailability of vasodilators such as nitric oxide (NO) is decreased, and endothelium-derived contractile factors are increased, and this phenomenon favours atherosclerosis [11]. The genetic transfer of cardiovascular diseases from one generation to the next may occur through epigenetic modifications caused by environmental exposures, especially concerning diet this is a major factor that has been implicated in cardiovascular disease proliferation is a high-fat diet, whose effect can be felt in almost every physiological system [12].

Oxidative stress and inflammation are the mechanisms through which high-fat diets accelerate the progression of cardiovascular disease [13]. Reports have revealed that increase in lipoprotein levels induces changes in the arterial system which aggravates the incidence of atherosclerosis and cardiovascular disease in adulthood [14]. Elevated total cholesterol (TC), low-density lipoprotein cholesterol (LDL-c), and triglycerides (TG) levels, as well as low high-density lipoprotein cholesterol (HDL-c) concentrations present during dyslipidemia, are known cardiovascular risk factors [14].

Also, the need for proper diagnosis of cardiovascular diseases has given preference to values of lipid ratios, TG: HDL-c, LDL-c: HDL-c, and TC: HDL-c ratios have become strong metabolic predictors for cardiovascular disease [15]. The Atherogenic index of plasma (AIP) obtained from the ratio of triglycerides to high-density lipoprotein is a novel marker of dyslipidemia and associated cardiovascular diseases [16–19]. An increase in AIP is associated with obesity and can be used clinically to quantify the effect of hypolipidemic drugs on the management of obesity, giving an idea of how to control the negative effect of long-term use of lipid-lowering drugs [14].

It has been noted that to overwhelm obesity, properly designed calorie restrictions and programs that are enhanced by behavioural therapy may not be sufficient; therefore, to achieve an effective and reliable anti-obesity therapy with very minimal side effects, potential herbs are constantly being investigated. [20]. Testing potential anti-obesity drugs' efficacy has given rise to many established experimental models. But the most outstanding one that provides an accurate picture of human obesity is the diet-induced obesity model, which is usually carried out with a high-fat diet [21]. Previous reports also indicated that the use of rats is more appropriate for monitoring body weight change which is a major indicator of obesity [22]. Evidence from previous studies showed that treatment of high-fat- diet-fed rats with extracts of *Gnidia glauca* [23], *Morinda citrifolia* [24]*, Anthocleista vogelii* [25] *and Silybum marianum* [26] displayed strong anti-obesity activities by reducing organo-somatic and atherogenic indices. Therefore justifying the use of this animal model for this study. However, there are still numerous plants with anti-obesity potentials that are employed in folk medicine that are yet to be investigated. Sweet melon belongs to this category.

Sweet melon, botanically identified as *Cucumis melo* and also known as golden melon, is a sizeable melon with a pale green to white succulent, juicy, sweet inner flesh and a bright golden-coloured skin [27, 28]. Sweet melon is a member of the family of Cucurbitaceae (Cucurbit) [28]. *Cucumis melo* is rich in essential nutrient and minerals which may include magnesium, potassium, iron, vitamin C, vitamin A, vitamin B6, calcium, pantothenic acid, zinc, omega-3, and omega-6. Regulation of blood pressure which is majorly mediated by the presence of potassium has been associated with sweet melon intake [29].

*Cucumis melo* has an excellent constituent of both soluble and insoluble fibre, and the high level of dietary fibres present in this fruit has been suggested to be very effective in preventing constipation and enhancing easy digestion of food. Essentially, bad LDL-c and other toxins are flushed out of the body by the action of *Cucumis melo* [30].

Lee et al*.* [31] demonstrated the therapeutic effect of *Cucumis melo L* in mice. In their study, *Cucumis melo* ameliorated insulin resistance and inflammation in the adipose tissue of obese mice. They concluded that *Cucumis melo* is a promising plant in the management of insulin resistance and adipose inflammation occurring due to obesity. Similarly, the anti-obesity ability of *Cucumis melo* has been tested in humans. Oral administration of *Cucumis melo* improved obesity and diabetes through an anti-inflammatory mechanism and without side effects [32]. The authors reported a significant decrease in fat weight, body weight, and size of adipocytes of subjects following four-week, oral exposure to *Cucumis melo*. Additionally, these authors tested *Cucumis melo* in mice and observed that it improved glucose metabolism by lowering the high-fat-diet-induced increase in fasting insulin, fasting blood glucose, and insulin resistance in a dose-dependent manner. It also increased insulin receptor gene expression and improved lipid profile. Also, Cucumis *specie agrestis var. agrestis* extract reduced the concentrations of triglycerides, LDL-c, and TC in high-fat diet dyslipidemic hamsters. It also enhanced the expression of mRNA APOA1 and LCAT, which are genes responsible for the reversal of cholesterol transport. Further, it increased hepatic mRNA expression of LXR α thus inhibiting lipogenesis [32]. However, the cardioprotective effects of *cucumis melo* is not fully understood. Therefore, this study which focuses on the anti-atherogenic and cardio-protective effect of Methanolic extract of *Cucumis melo* seeds on high fat-diet induced obesity in male Wistar rats becomes imperative.

## Methods

### Animals

Forty (40) male Wistar rats showing no symptoms of any disease with body weights ranging from 120—150 g were procured from The National Veterinary Research Institute, Vom, Jos. The animals were cared for in the Bingham University Central Animal house, acclimatized for two weeks, and given free access to feed and water ad libitum. Vital Feeds, manufactured by UAC, Jos, Nigeria, purchased from Nasarawa State distributor, were given under standard laboratory conditions of temperature (37.5–37.8 oC), humidity (55 ± 15%) with 12 h light and dark cycle. Afterwards**, **they were randomly grouped into 5 using their weight (*N* = 8) as follows;

- Group 1- control (distilled water, rat chow).
- Group 2- fed with the high-fat diet (HFD).
- Group 3- HFD + 50 mg/kg of methanolic extract of *Cucumis Melo.* Seeds (MCMs).
- Group 4- HFD + 100 mg/kg of MCMs.
- Group 5- HFD + 200 mg/kg of MCMs.

To decide the dosage regimen as above the median lethal *dose* (LD50) was carried out following established method [33], no mortality was observed, and the lowest effective dose of 50 mg/kg was chosen followed by 100 mg/kg and 200 mg/kg as also reported by Shahana et al., (2020) [34].

The animals were treated according to the standards of the National Institutes of Health (NIH) guide for taking care of laboratory animals (NIH Publications No. 8023, revised 1978). Procedures and animal use were endorsed by the Faculty of Basic Medical Sciences Research Ethics Review Committee of Ladoke Akintola University, Ogbomoso, Nigeria. [FBMS/RERC/05/2022; Dated: 12/2/2022].

### Experimental protocols

#### Preparation of the High Fat Diet (HFD) and induction of obesity

The pellet of a normal diet has 5% fat, 30% protein, and 60% carbohydrates, and the high-fat diet (HFD) contains 50% fat, 25% carbohydrates, and 20% protein. The HFD was formulated using various classes of food which include: carbohydrates (cassava locally processed into *garri*), protein (dried Bonga fish), and fat (butter), according to the method of Onyeneke and Anyanwu [35]. The quantity of fibre, minerals, and vitamins for normal and high-fat diets was the same.

Group I rats received normal diet while group II-V rats received high-fat diet for a period of 10 weeks to induce obesity, and groups III-V were treated with graded doses of extract for two weeks to evaluate the effect of MCMs on obesity-induced with diet. Treatment was done by two assigned scientists at 7; 30am daily. The lead investigator was the only one aware of the treatment given to the groups throughout the study.

#### Plant authentication and preparation of extract (Methanolic Extraction)

The fruit of *Cucumis melo* was obtained from Zaria-road fruit market, Jos. The collection of the plant adhered to the guidelines stipulated by the National Institute for Pharmaceutical Research and Development, the plant was then was identified by Akeem A. Lateef, a Taxonomist at the Herbarium and Ethnobotany Unit, Department of Medicinal Plant Research and Traditional Medicine, National Institute for Pharmaceutical Research and Development. Idu-Abuja, Nigeria. The plant specimen was deposited at the herbarium with a Voucher specimen number -NIPRD/H/7216.

The seeds were separated from the fruit, washed, and air-dried at room temperature. The seeds were pulverized with an electric blender and air-dried. The constituent of the air-dried seed (200 g) was extracted and defatted using N-hexane (boiling point: 40–60 °C) in a soxhlet extractor for 24 h. The defatted, dried marc was repacked and extracted with methanol for ten hours. The concentrated methanol extract was diluted to twice its volume with distilled water and further sieved to separate the shaft, after which it was exposed to air for the volatile methanol to escape totally. The studies of Mallek-Ayadi et al. (2018) [36] supported the use of methanolic extraction, it confirmed the presence of high oil content in the seeds of cucumis melo. In addition the yield with methanol was higher and still retains the antioxidant properties of the seed.

#### Qualitative and quantitative phytochemical screening

QuALITATIVE Phytochemical assessment was executed following methods described by other investigators [37, 38]. The following phytochemicals were tested for: Saponin, Terpenoid, Phenolics, Coumarin, Tannin, Glycosides, Steroid, Alkaloids, Flavonoid, Anthocyanin, AminoAcid, phlobatanin, Carbohydrate, and Triterpene.

Quantitative screening of the identified phytochemicals was carried out using previous method [39].

#### GC–MS analysis

GC–MS analysis of MCMs was executed with an Agilent 5977B GC/MSD system coupled with Agilent 8860 auto-sampler, a Gas Chromatograph interfaced to a Mass Spectrometer (GC–MS) fitted with an Elite-5MS (5% diphenyl/95% dimethyl polysiloxane) fused a capillary column (30 × 0.25 μm ID × 0.25 μm df) because of GC–MS detection, a system of electron ionization was employed in electron impact mode using an ionization energy of 70 eV. A carrier gas (Helium gas = 99.999%) was put at a constant flow rate of 1 ml/min, and an introductory volume of 1 μl was employed at a split ratio of 10:1. A Five point serial dilution calibration standards (1.25, 2.5, 5.0, 10.0 ppm) were constituted from the stock solution of 40 ppm and employed in the calibration of the GC–MS.

A 300 °C injection temperature was sustained, and the temperature of the ion-source was 250 °C, and that of the oven was mapped out from 100 °C (isothermal for 0.5 min), in company of an increase of 20 °C/min to 280 °C (2.5 min), Mass spectra were set at 70 eV; with a scanning interval of 0.5 s and fragments from 45 to 450 Da. The solvent delay was 0 to 3 min, and the total GC/MS conduction time was 12 min [40].

#### Assessment of food and energy intake

Assessment of food and energy intake was carried out according to previous method [22] with modifications, The animal’s body weight and food intake was measured once a week during the study. The diet intake in each cage was calculated as:

$$Total\;food\;in\;take\;(Wd)=(initial\;food\;weightW0)-W1\;(left\;over\;food\;weight)-W2(spilled\;food\;weight)$$

The spilled food was weighed after feces had been separated and spilled food had been dried.

$$Energy\;in\;take\;was\;measured\;as:\;Energy=wd/7/n\ast Et$$

$$n=number\;of\;rats,.ET=total\;energy\;of\;normal\;rat\;diet\;or\;total\;energy\;of\;high\;fat\;diet\;in\;HFD\;groups$$

$$Energy\;of\;normal\;rat\;pellet=16.1KJ/g,Energy\;of\;High\;Fat\;diets=22.1KJ/g$$

#### Measurement of anthropometric parameters

This was done according to the previous method [41]. The weekly body weight was measured in grams (g). The length of the body (nose-to-anus length) was also determined weekly and measured in centimetres (cm). The length and body weight were utilized in the calculation of body mass index (BMI). Body mass index (BMI) = body weight (g)/length2 (cm2).

#### Measurement of adipose tissue and fat mass determination

Animals were sacrificed at the end of the treatment period by euthanasia, making use of ketamine (4 mg/kg b.w.) and xylazine (40 mg/kg b.w.) injection intraperitoneal. The measurement of adipose tissue and fat mass determination was performed according to the standard method. [25]. Following the incision of the abdomen, white adipose depots from five different sites (two subcutaneous and three intra-abdominal) and interscapulum brown adipose tissue were harvested from each rat and were dried on separate filter papers; their weights in grams (g) were recorded. The total fat mass was determined as the addition of weights of white adipose tissue and brown adipose tissue, while the relative weight of fat tissue was obtained by dividing total fat mass by the body weight.

#### Measurement of adiposity index

The adiposity index was done according to the method described by previous researchers [42]. The isolation of adipose tissue was performed from the epididymis, visceral and retroperitoneal pad. The isolated adipose tissues were dried on filter paper, and their weight (g) was recorded. To determine the adiposity index the sum of the epididymis, visceral, and retroperitoneal fat weights divided by body weight, multiplied by 100, was expressed as adiposity index in percentage.

#### Measurement of serum glucose

Blood was collected via cardiac puncture, serum was obtained through centrifugation, and aliquots were taken to measure the concentration of glucose using commercial kits from Labtest Diagnostic S.A (Minas Gerais, Brazil). The serum glucose was measured according to the method described previously [43].

#### Preparation of tissue homogenate

The animal was dissected, and the heart was harvested. The heart was washed in distilled water, and the weight was measured. Thereafter, the heart tissue was homogenized. Samples were in 4 volumes of 5 mM phosphate buffer, pH 7.4, and centrifuged at 10,000 revolutions for 15 min to obtain clear supernatant, which was stored at -80 °C.

#### Measurement of lipid profile

The level of Cholesterol and Triglyceride in the serum was measured using the GPO-PAP-enzymatic colourimetric method [44]. High-density lipoprotein (HDL) was determined by the precipitation method and Low-density lipoprotein cholesterol was determined by the method described by Friedewald et al. in the year 1972 [45].

#### Determination of atherogenic indices

The cut-off by the expert panel of the National Cholesterol Education Programme (NCEP) (2002) [46] was the basis on which the plasma lipid abnormality was determined. Further, atherogenic indices such as Castelli’s Risk Index-I (CRI-I) = TC/HDL-c, Atherogenic Coefficient (AC) = (TC– HDL-c)/HDL-c, TG/HDL-c ratio, Atherogenic Index of Plasma (AIP) = log (TG/HDL-c) are calculated from each group**.**

#### Measurement of protein content

The concentration of protein in samples collected was determined using the Biuret method as previously described [47]. A slight modification was made to this method which is the addition of potassium iodide to the reagent to prevent precipitation of Cu2 + ions as cuprous oxide.

#### Assessment of lipid peroxidation

The lipid peroxidation assessment was carried out by measuring the thiobarbituric acid reactive substances (TBAR) produced during Lipid peroxidation. A chromogen produced from the reaction of thiobarbituric acid and malondialdehyde was observed at 531.8 nm. This was carried out using the method of Varshney and Kale (1990) [48].

#### Measurement of catalase activities

To determine the catalase activity, the method of Farmand (2005) [49] was used. This method depends on the loss of absorbance observed at 240 nm as catalase splits hydrogen peroxide.

#### Determination of total nitrite

The method reported by Ignarro in the year 1989 [50] was used for the process of nitrite determination. Diazotization reaction, which was described previously [51], is the principle this assay depends on. The method is based on the chemical reaction which uses sulfanilamide and naphthyl-ethylenediamine dihydrochlorate (NED) under acidic conditions. Sulfanilamide and NED compete for nitrite in the Griess reaction.

#### Statistical analysis

The mean and standard deviation (SD) of all values were calculated. The results expressed as Mean ± SD were subjected to one-way ANOVA using graph pad prism version 5.0, followed by Bonferonni ‘s post hoc test. The level of significance was tested at *P* ≤ 0.05.

## Results

### Qualitative and quantitative phytochemical screening

The result of phytochemical screening was positive for Terpenoids, phenolics, Glycosides, Steroids and carbohydrate. Steroid has the highest quantity (19.80 mg/100 g), followed by phenolics (4.05 mg/100 g), carbohydrate (2.23 mg/100 g), then glycosides (0.10 mg/100 g) and the least was terpenoid (0.05 mg/100 g), as shown in Table 1.

**Table 1:** Qualitative and Quantitative analysis of phytochemicals in MCMs

Phytochemical	Terpenoid	Glycosides	Steroid	Carbohydrate	phenolics
Qualitative analysis Presence ( +)/ absence (-)	** + **	** + **	** + **	** + **	** + **
Quantitative analysis (mg/100 g)	**0.05**	**0.10**	**19.80**	**2.23**	**4.05**

### GC–MS results

**Fig. 1:**
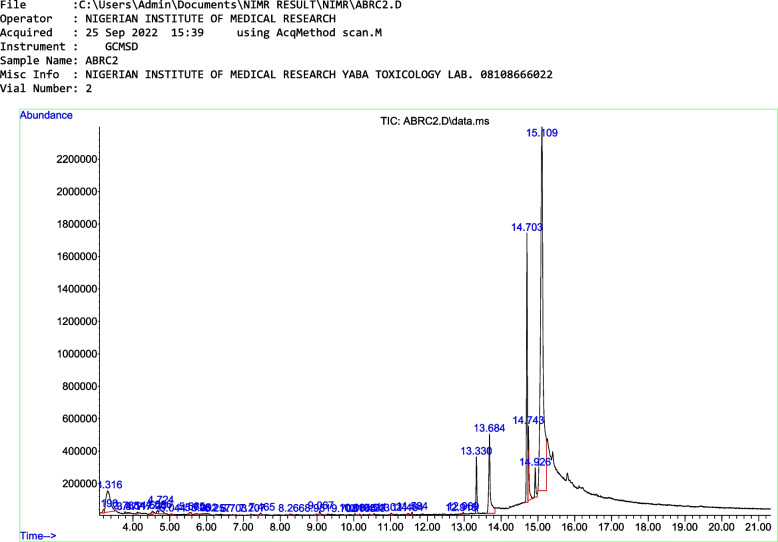
GC–MS Chromatogram of *Cucumis melo*. L. Inodorus

  The Interpretation of mass spectrum GC–MS was conducted using the database of the National Institute Standard and Technology (NIST) which have more than 62,000 patterns and the National Center for Biotechnology Information. The spectrum of the unknown components was compared with the spectrum of known components stored in the NIST library. The name of the 35 compounds, their retention time and area are shown in Table 2 below. Figure 1 below also shows the peak time of the individual components.

**Table 2:** Name, retention time and area of identified compounds

S/N	RT	AREA	COMPOUND
1	3.196	0.33	Butane, 2,2’-thiobis-
2	3.316	5.69	L-Arabinitol
3	3.785	0.12	2-Furanmethanamine, N-methyl-
4	3.877	0.16	(5-Methylcyclopent-1-enyl)methanol
5	4.117	0.17	Imidosulfurous difluoride, methyl-
6	4.523	0.34	Isoquinoline, 1,2,3,4-tetrahydro-
7	4.666	0.29	Undecane
8	4.724	1.04	Benzoic acid, methyl ester
9	5.044	0.09	1-(2-Adamantylidene)semicarbazide
10	5.565	0.30	Benzene, pentyl-
11	5.725	0.08	Sulfonium, (acetylamino)diethyl-
12	5.902	0.06	2-Naphthalenol, 1,2-dihydro-, acetate
13	6.257	0.08	trisiloxane, 1,1,1,5,5,5-hexamethy l-3-[(trimethylsilyl)oxy]-
14	6.703	0.06	Cyclopropane, 1,1-dimethyl-2-(2-propenyl)-
15	7.207	0.11	p-Cymen-7-ol
16	7.465	0.24	2-Methoxy-4-vinylphenol
17	8.266	0.08	Cyclopentasiloxane, decamethyl-
18	8.981	0.10	Eugenol
19	9.067	0.26	1-Hexanone, 1-phenyl-
20	9.708	0.07	1,3-Di-O-acetyl-.alpha.-.beta.-d-ribopyranose
21	10.034	0.11	1,3,5,7,9-Pentaethylcyclopentasiloxane
22	10.165	0.09	formamide, N-methyl-N-(4-nitrophenyl)-
23	10.423	0.09	m-Tolyl isothiocyanate
24	10.543	0.07	Tetrahydrofuran-2-one, 5-[1-hydroxyhexyl]-
25	11.024	0.17	3-Heptyne
26	11.464	0.09	(E)-2,6-Dimethoxy-4-(prop-1-en-1-yl)phenol
27	11.584	0.16	Ethyl 4-hydroxyphenylacetate, TBDMS derivative
28	12.918	0.06	Tricyclo[5.4.3.0(1,7)]tetradecane-3,6-diol, 4-formyl-2,4,7,14-tetram ethyl-, diacetate
29	12.969	0.10	N-Methyladrenaline, 3TMS derivative
30	13.330	3.02	Hexadecanoic acid, methyl ester
31	13.684	7.45	n-Hexadecanoic acid
32	14.703	14.87	9,12-Octadecadienoic acid (Z,Z)-, methyl ester
33	14.743	3.86	11-Octadecenoic acid, methyl ester
34	14.926	1.74	Heptadecanoic acid, 16-methyl-, methyl ester
35	15.109	58.44	9,12-Octadecadienoic acid (Z,Z)-

### Effect of MCMs on food and energy intake

**Fig. 2:**
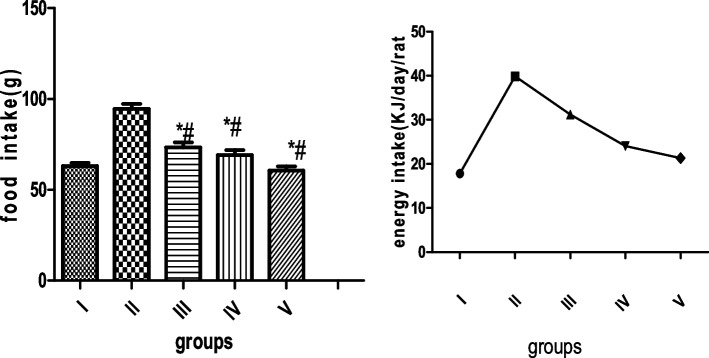
Effect of MCMs on food and energy intake. All values are expressed as mean ± SD, *P* < 0.05, (*) significant compared with group I, (#) significant compared to group II

Food intake significantly increase (*P* < 0.05) in HFD compared to groups treated with MCMs. While, energy intake decreased significantly (*P* < 0.05) in the MCMs administered groups compared to the HFD groups. The results are presented in Fig. 2

### Effect of MCMs on body weight, body fat, and adiposity index

Animals fed with the high-fat diet only have b.w., BMI, brown fat, white fat, fat mass, and adiposity index that were significantly (*p* < 0.05) higher than the control group. White fat, fat mass, and adiposity index in MCMs treatments were also significantly (*p* < 0.05) higher compared to the control group.

While administration of 50, 100, and 200 mg/kg of MCMs significantly (*p* < 0.05) decreased BW, BMI, brown fat, white fat, fat mass, adiposity index and relative weight of fat mass when compared to the HFD-only group with 100 mg/kg of MCMs giving the lowest values as shown in Table 3.

**Table 3:** Effect of MCMs on body weight, body fat and adiposity index

Parameter /groups	I	II	III	IV	V
B.W (g)	**146.30 ± 0.88**	196.00 ± 3.46*	**138.80 ± 8.31#**	**141.7 ± 5.89#**	**153.20 ± 7.48#**
BMI (g/m^2^)	**0.27 ± 0.01**	0.40 ± 0.01*	0.36 ± 0.02*#	**0.24 ± 0.01#**	**0.28 ± 0.03#**
Brown fat(g)	**0.56 ± 0.05**	0.81 ± 0.03*	**0.57 ± 0.03#**	**0.69 ± 0.21**	0.81 ± 0.03*
White fat(g)	**0.92 ± 0.01**	3.87 ± 0.05*	2.67 ± 0.23*#	2.36 ± 0.01*#	2.75 ± 0.08*#
Fat mass (g)	**1.49 ± 0.06**	4.68 ± 0.05*	3.24 ± 0.26^*#^	3.05 ± 0.02*#	3.56 ± 0.09*#
Adiposity Index	**1.31 ± 0.06**	3.82 ± 0.45*	2.49 ± 0.03*#	2.39 ± 0.26*#	2.87 ± 0.26*#
**RW of Fat mass (g/Kg)**	**0.019 ± 0.00**	0.062 ± 0.01*	0.043 ± 0.00*#	0.035 ± 0.01*#	0.030 ± 0.01*#

All values are expressed as mean ± SD, *P* < 0.05, (*) significant compared with group I, (#) significant compared to group II

*RW* Relative Weight

### Effect of MCMs on lipid profile

**Fig. 3:**
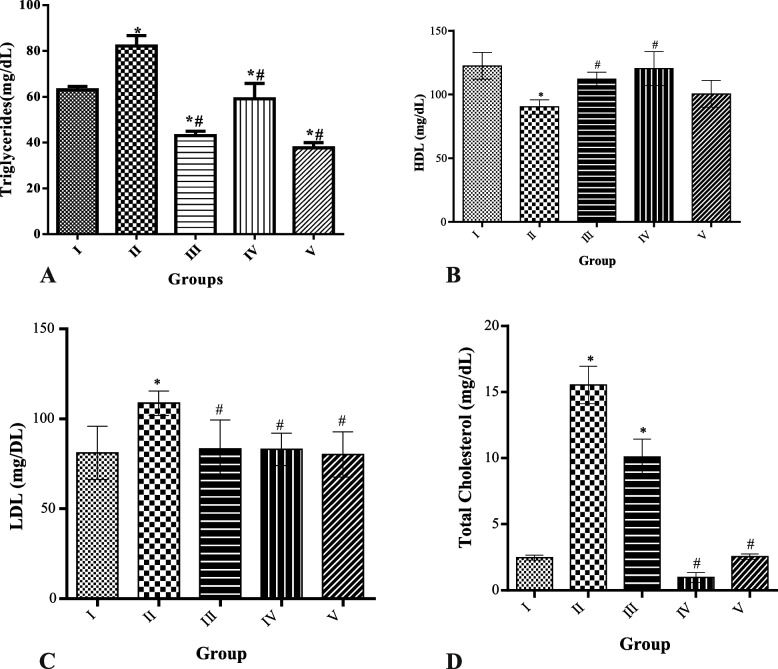
Effect of MCMs on lipid profile. 3A = triglyceride, 3B = HDL, 3C = LDL, 3D = Total cholesterol. All data are represented as Mean ± SD, *P* < 0.05. * Significant as compared with control, # significant compared with group II

Significant (*p* < 0.05) increases in serum triglycerides, LDL, and total cholesterol were observed in HFD-only rats compared to the control group. Also, a decrease in HDL-cholesterol was observed in HFD-only rats compared to the control group. Administration of 50, 100, and 200 mg/kg MCMs significantly (*p* < 0.05) decrease the serum triglycerides, LDL, and total cholesterol compared to the HFD-only animals with 100 mg/kg MCMs treatment having the lowest value of total cholesterol. HDL in the MCMs treated groups showed a significant (*p* < 0.05) increase compared to the HFD-only rats as shown in Fig. 3(A-D).

### Effect of MCMs on cardiovascular risk indices

**Fig. 4:**
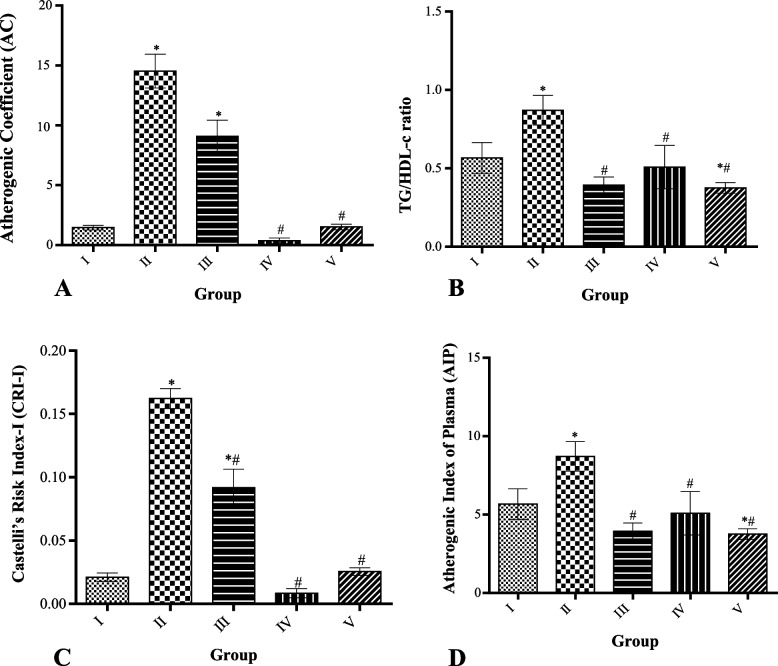
Effect of MCMs Cardiovascular Risk Indices. 4A=Atherogenic Coefficient (AC), 4B=TG/HDL-c ratio, 4C=Castelli's risk index-I (CRI-I). 4D= Atherogenic of index of plasma All data are represented as Mean ± SD, *P* < 0.05. * Significant as compared with control, # significant compared with group II

TG/HDL-C ratio, Atherogenic Index of Plasma, Atherogenic Coefficient, and Castelli’s risk index were significantly (*P* < 0.05) increased in HFD-only groups compared to the control. Treatment with 50, 100, and 200 mg/kg MCMs significantly (*p* < 0.05) decreased all the indices compared to the HFD-only group, with 100 mg/kg of MCMs having the lowest value for Atherogenic Coefficient, and Castelli’s risk index as shown by Fig. 4 (A-D).

### Effect of MCMs on the weight of the heart

**Fig. 5:**
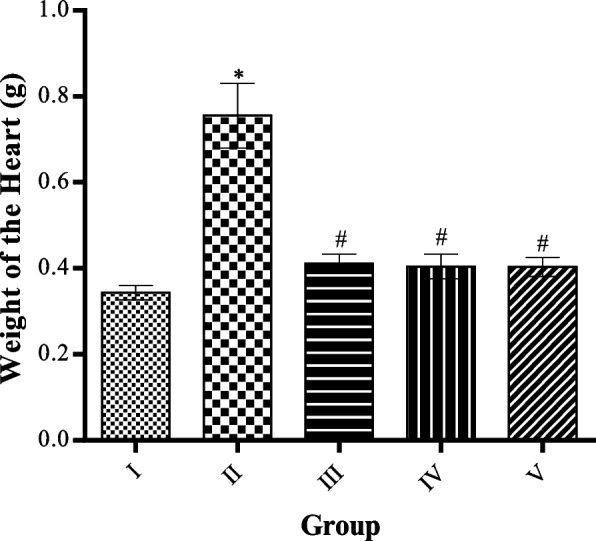
Effect of MCMs on Weight of the Heart. All data are represented as Mean ± SD, *P* < 0.05. * Significant as compared with control, # significant compared with group II

A high-fat diet only significantly (*p* < 0.05) increased the weight of the heart compared to the control group. While administration of MCMs at doses of 50, 100, and 200 mg/kg body weight significantly (*p* < 0.05) reduced the weight of the heart compared to the HFD-only group as shown in Fig. 5.

### Effect of MCMs on serum protein level

**Fig. 6:**
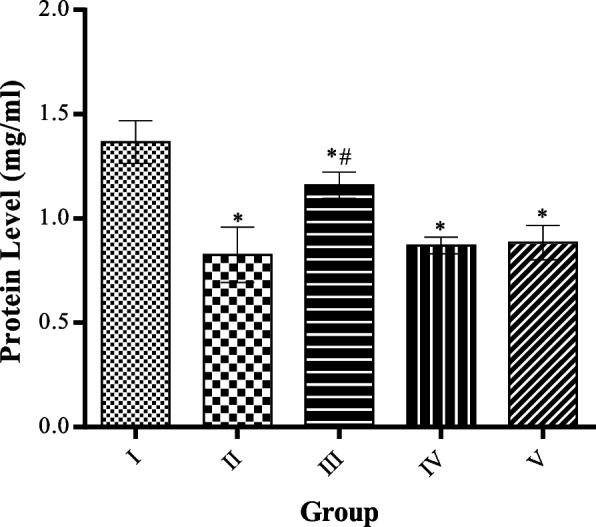
Effect of MCMs on Protein Level. All data are represented as Mean ± SD, *P* < 0.05. * Significant as compared with control, # significant compared with group II

The serum protein level in the high fat-diet rats with or without MCMs treatment was significantly reduced compared to the control group while administration of 50 mg/kg of methanolic extract of *Cucumis Melo.* Seeds significantly (*p* < 0.05) increased serum protein levels compared to the high fat-diet only group, as shown in Fig. 6.

### Effect of MCMs on malondialdehyde level in cardiac tissue homogenate

**Fig. 7:**
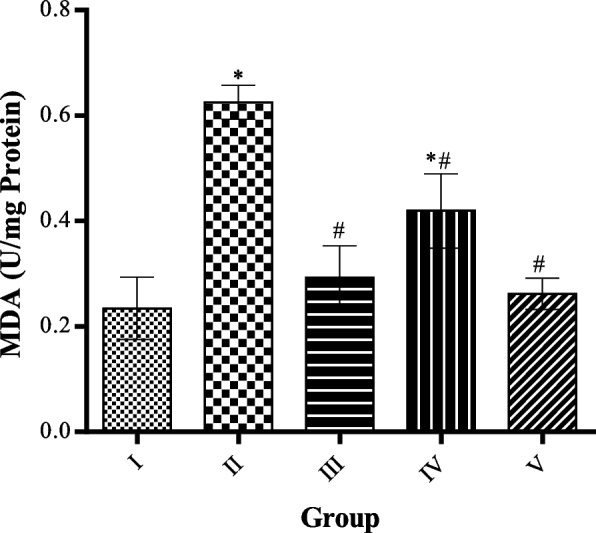
Effect of MCMs on Malondialdehyde. All data are represented as Mean ± SD, *P* < 0.05. * Significant as compared with control, # significant compared with group II

MDA level was significantly (*p* < 0.05) increased in the heart tissue homogenate of HFD only and HFD rats fed with 100 mg/kg MCMs compared to the control group. MCMs administered at 50, 100, and 200 mg/kg body weight significantly (*p* < 0.05) decreased MDA level compared to HFD untreated group as shown in Fig. 7.

### Effect of MCMs on catalase activities in cardiac tissue homogenate

**Fig. 8:**
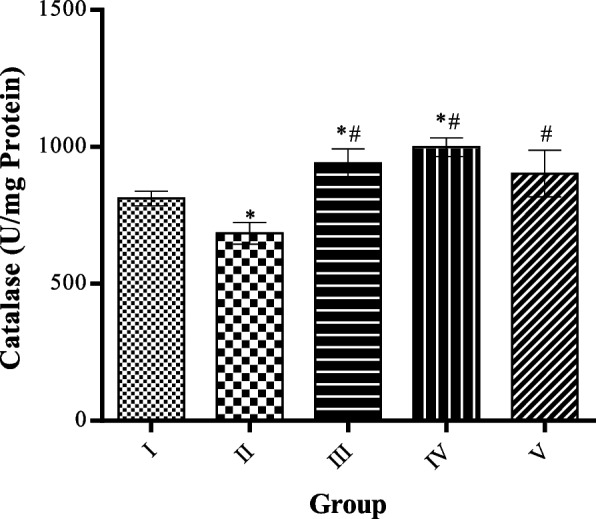
Effect of MCMs on Catalase. All data are represented as Mean ± SD, *P* < 0.05. * Significant as compared with control, # significant compared with

The level of catalase significantly (*p* < 0.05) decreased in HFD only compared to the control group. While catalase significantly (*p* < 0.05) increased in the groups treated with MCMs compared to the HFD untreated group as shown in Fig. 8.

### Effect of MCMs on nitrite level in cardiac tissue homogenate

Nitrite level was significantly (*p* < 0.05) decreased in HFD only cardiac tissue homogenate.

**Fig. 9:**
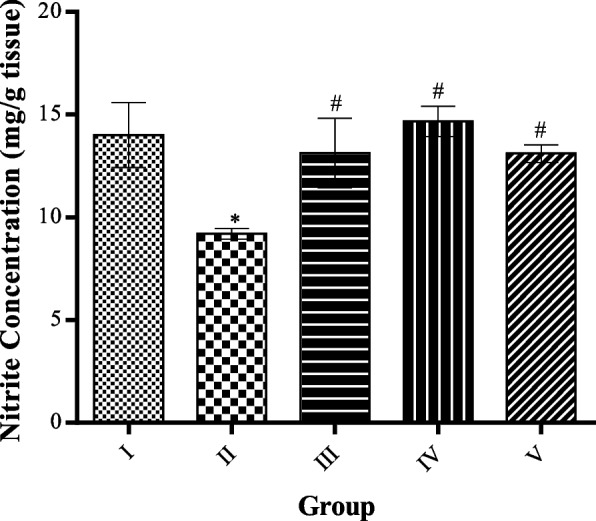
Effect of MCMs on Nitrite Level of Plasma. All data are represented as Mean ± SD, *P* < 0.05. * Significant as compared with control, # significant compared with group II

Compared to the control group, nitrite levels significantly (*p* < 0.05) increases in the MCMs treated groups compared to the HFD-only group, especially the 100 mg/kg treatment, this is reported in Fig. 9.

## Discussion

The objectives of this study were to assess the anti-atherogenic and cardio-protective effects of Methanolic extract of *Cucumis melon* Seeds on a high-fat diet (HFD)-induced obese rats. When the energy intake of an individual exceeds the energy output, and there is an overall increase in weight, enlarged fat mass, and increased lipid concentration in the blood, a condition known as obesity ensues. The intake of food and the weight of the body are direct measures of obesity [52]. The decrease in food and energy intake in rats fed with MCMs supports the ability of sweet melon extract for anti-atherogenic and cardio-protective properties observed in this study.

The increased BW, BMI, brown fat, white fat, fat mass, adiposity index and relative weight of fat mass in response to a high-fat diet observed in this study agrees with previous reports on animals fed with high-fat diets, this response is due to the ability of high fat-diet to increase the energy intake than the expenditure [53]. However, MCMs significantly decreased BW, BMI, brown fat, white fat, fat mass, and adiposity index in HFD-fed rats during the treatment period especially as seen in week 10. This effect is in order with a previous study in which *Cucumis Melo. L* decreased the BW in obese humans and HFD-fed mice [32]. HFDs significantly increase the cardiac weight in this study. This is in tandem with the previous study [54] that reported increased cardiac weight in rabbits following HFDs.

The BMI is an important screening tool to detect overweight and obesity, the high BMI in HFD shows that the rats were obsessed and the decrease in BMI following administration of extract lay credence to the ability of the extract to manage obesity. This can be compared with similar work on ethanol leaf extracts of *Aegle Marmelos* which substantially cause decrease in the body weight and BMI of HFD-fed rats [55]. At the cellular level, obesity is characterized by an increase in the population and size of adipocytes that are differentiated from fibroblasts in adipose tissues to compensate for the need to store extra lipids [56], this makes excessive or abnormal fat deposition and accumulation in the adipose tissue a common occurrence in obesity [25]. Therefore, the assessment of fat mass in experimental animals is relevant in obesity studies. The decrease in adiposity index following MCMs treatment is similar to results obtained in previous studies [25, 57]. It has also been reported that sweet melon seed possesses both soluble and insoluble fibre [30], which must have altered the deposition of fats on some reference organs used in determining the adiposity index and could be responsible for the decreased adiposity index.

Significant increases in serum triglycerides, LDL and total cholesterol, and decreased HDL were observed in HFD obese rats in this study. These observations were similar to previous studies on HFD [58, 59] and could be linked to the fact that HFD increased lipid metabolism in the liver and accumulation of lipid in the blood known as hyperlipidemia a known risk factor that contributes to coronary heart disease [60]. But, the administration of methanolic extract of Cucumis melon reduced serum triglycerides, LDL, total cholesterol, and increased HDL levels which suggests that the extract could improve lipid metabolism as observed for watermelon [61]. The reduction of LDL cholesterol levels is necessary for obesity to treat and also prevent various complications that might arise [62].

It has been illustrated that lipid ratios such as the Castelli's risk index-I (CRI-I), CRI-II, atherogenic coefficient (AC), CHOL Index, and the atherogenic index of plasma (AIP) are now important in the prognosis of cardiovascular risk development [63–65]. These indices were raised following HFD, but treatment with 50, 100, and 200 mg/kg MCMs significantly (*p* < 0.05) decreased all the indices compared to the HFD-only group, with 100 mg/kg of MCMs displaying the lowest value for Atherogenic Coefficient, and Castelli’s risk index. It has been revealed from studies that the TG/HDL-C atherogenic index evaluates the presence of insulin resistance and alteration in an obese individual [66, 67]. The atherogenic indexes are very important in determining the risk of cardiovascular disease in an obese individual. It shows precipitation of fatty acids /lipids in the heart’s large vessels (coronaries, aorta) and in the kidney, the report suggested the increase in atherogenic indexes is a great risk for oxidative damage in the aforementioned organs [15]. The results of the lipid profile and the atherogenic indices suggest that they possess cardioprotective and anti-atherogenic properties which is the first study to do so.

HFDs increase the risk of oxidative stress and had been associated with the etiopathogenesis of cardiovascular diseases. Malondialdehyde which is a reactive substance in thiobarbituric acid is used in the determination of lipid peroxidation in tissues and is a significant marker of oxidative stress [68]. MDA level was significantly increased in the heart of HFD compared to the control, *P* < 0.05. Administration of MCMs significantly decrease the MDA level in the heart of HFD-fed rats compared to HFD untreated rats, *P* < 0.05. Reports have shown that plants contain free radicals scavenging molecules such as phenols, flavonoids, vitamins, and terpenoids that are rich in antioxidant activity [69, 70]. It is known that Antioxidants are effective against the harmful effects of reactive oxygen species on cells and tissues [68, 71]. In the maintenance of health and prevention of disease conditions, natural antioxidants played an important role [72]. Studies have documented the phytochemical screening of methanolic extracts of *Cucumis melo* L. seeds to contain alkaloids, flavonoids, phenolics, steroids, cardiac glycosides, and terpenoids with powerful free radical scavenging activities [73]. The presence of glycoside presents a possible intrinsic action to inhibit the incidence of heart failure and arrhythmia through a mechanism suggested to be via up regulation of ca2 + and inhibiting Na + /K + pump so that it enhances contractility of the cardiac muscle which enhances the pumping action of the heart [74]. Theses phytochemicals also act as antioxidants and could protect the heart by inhibiting the formation of free radical in which terpenoid have also been implicated [75]. Therefore the presence of glycoside, phenolics, terpenoid and steroids could be implicated in the anti-atherogenic and cardio-protective properties of the seed extract. The GCMS also confirmed an array of compounds that could be implicated in this anti-atherogenic and cardio protective properties and needed further studies for their medicinal potentials.

NO causes the activation of soluble guanylyl cyclase (GC) leading to increase in the intracellular cyclic guanosine monophosphate (cGMP) levels that regulate relaxation of the vascular smooth muscle and inhibit platelet aggregation and adhesion [76]. In this study, nitrite level was significantly increased in the MCMs treated especially the 100 mg/kg, and 200 mg/kg compared to the HFD and control, *P* < 0.05. HFDs are reported to inhibit NO synthesis [77]. However, the administration of MSM activities was in tandem with the previous report [78]. The results from this present study showed that the methanolic extract of sweet melon seeds (*Cucumis melo*. L. Inodorus) prevents obesity, protects against hyperlipidemia, improves cardiac risk indices, catalase activities, protein level, nitrate concentration and reduces oxidative stress that could result from consumption of a high-fat diet.

## Conclusion

*Cucumis melo*. L. Inodorus sweet melon has shown enormous potential in the treatment and management of atherogenic and cardiovascular disease conditions through the amelioration of disease risk factors. It significantly reduced TG, TC, LDL, weight, body mass and adiposity index, atherogenic index, Castelli risk index, AIP, Cardiac redox status, and the nitrite level and enhances the activity of catalase antioxidant enzyme in HFD-induced obese rats at 100 mg/kg b.w. and 200 mg/kg b.w. compared with the normal and against the disease control groups. Hence, it is credible to suggest that MCMs has anti-atherogenic property, possibly associated with the presence of phenolics, glycosides and steroids which have high antioxidant and hypocholesterolemic properties. A daily dose of 100 mg/kg may be recommended for rat and a dose of 16.21 mg/kg in human from the result of this study. However, further study is needed to evaluate the molecular mechanisms to which these anti-atherogenic actions can be attributed, and evaluate the compounds identified through the GCMS results with the aim of developing drug candidates in the management of obesity and its complications.

## Data Availability

The datasets generated during and/or analysed during the current study are available from the corresponding author on reasonable request.

## Abbreviations

AC: Atherogenic coefficient
AIP: Atherogenic index of plasma
BMI: Body mass index
b.w.: Body weight
CAT: Catalase
cGMP: Cyclic guanosine monophosphate
CRI-I: Castelli's risk index-I
GC: Guanylyl cyclase
GPx: Glutathione peroxidase
HDL-c: High-density lipoprotein cholesterol
HFD: High-fat diet
LDL-c: Low-density lipoprotein cholesterol
MCMs: Methanolic extract of *Cucumis melon* seeds
MDA: Malondialdehyde
N: Normal
NED: Naphthyl-ethylenediamine dihydrochlorate
NO: Nitric oxide
ROS: Reactive oxygen species
SD: Standard deviation
SOD: Speroxide dismutase
TBARs: Thiobarbituric acid reactive substances
TC: Total cholesterol
TG: Triglycerides
